# TEACHING (WITH) FORTITUDE: ADDRESSING END-OF-LIFE AND ADVANCE CARE PLANNING THROUGH AN INTERDISCIPLINARY LENS

**DOI:** 10.1093/geroni/igae098.0882

**Published:** 2024-12-31

**Authors:** Tina Newsham, Alissa Dark-Freudeman, Jamy Chulak, Elizabeth Fugate-Whitlock, Kirsten Kringle-Baer

**Affiliations:** University of North Carolina Wilmington, Wilmington, North Carolina, United States; University of North Carolina Wilmington, Wilmington, North Carolina, United States; University of North Carolina Wilmington, Wilmington, North Carolina, United States; University of North Carolina Wilmington, Wilmington, North Carolina, United States; University of North Carolina Wilmington, Wilmington, North Carolina, United States

## Abstract

Topics related to end-of-life (EOL) and advance care planning (ACP) can be fraught, both for instructors and students. Further, teaching about these topics can be challenging as they can (and, we argue, should) be addressed through multiple disciplinary lenses, and ideally in an interdisciplinary fashion. In this paper, we share an interdisciplinary effort to teach about EOL and ACP at the University of North Carolina Wilmington. Faculty in Psychology, Gerontology, Philosophy, and Respiratory Therapy collaborated to create an interdisciplinary, seven-week module that was integrated into classes from each participating discipline. Students were grouped across disciplines and worked together to gather data regarding needs and desires related to EOL and ACP and then to create discussion guides around relevant topics to foster meaningful conversation and planning related to sensitive topics. Students completed guided reflections regarding what they learned about and from partners with different disciplinary perspectives and how the interdisciplinary collaboration resulted in outcomes that would otherwise have been absent in a singular discipline effort. This project serves as a model for interdisciplinary teaching, particularly around sensitive topics.

